# Assessing the resilience of portable vision tests to an uncontrolled home environment

**DOI:** 10.7717/peerj.20657

**Published:** 2026-02-20

**Authors:** Peter F. Reddingius, Mehal Rathore, David P. Crabb, Pete R. Jones

**Affiliations:** 1 Department of Optometry and Visual Science, City St George’s, University of London, London, United Kingdom; 2 Institute of Ophthalmology, University College London, London, United Kingdom

**Keywords:** Home-monitoring, Contrast sensitivity, Optometry, Ophthalmology, Telemedicine

## Abstract

**Background:**

In ophthalmology (and medicine more widely) there is increasing interest in telemedicine: having patients perform tests at home for greater efficiency and to meet growing demand. However, despite this increased interest in vision home monitoring, many vision tests are evaluated in standardised clinical settings, not home environments. Here, we investigated the resilience of two portable contrast sensitivity tests to the sorts of potentially confounding factors that may be encountered in a home setting.

**Methods:**

Normally sighted adults (*n* = 107) performed two contrast sensitivity tests (one pen-and-paper and one tablet-based). Testing took place in a furnished apartment, where we could control/measure various extraneous factors (including illumination, time of day, seating type, screen cleanliness). Key outcome measures were raw contrast sensitivity scores, test-retest repeatability, and test duration; and how these metrics varied with extraneous factors.

**Results:**

No effect of time of day, participant motivation, or seating type was observed (all *P_Bonferroni_* > 0.140). Scores on the pen-and-paper test were not affected by illumination (*P_Bonferroni_* = 0.348), except when tests were conducted in extreme darkness (≤1 lux; *P_Bonferroni_* = 0.036). A follow-up study indicated that screen smudging (caused by fingerprints) had no significant effect on the outcome of the tablet-based test (*P* = 0.573).

**Conclusion:**

Taken together, the results indicate that, contrary to our expectations, both digital and pen-and-paper contrast sensitivity tests appear relatively resilient to many of the sorts of extraneous factors encountered in a home setting. This speaks to the potential viability of vision home monitoring, though study limitations and necessary future work are discussed.

## Introduction

Many eye conditions are age-related, and so, as societies age (Tham et al., 2014; Wong et al., 2014), ophthalmology clinics worldwide are facing increasing strain (Foot & MacEwen, 2017; Foreman et al., 2020; Buys & Bellan, 2023). One way to help cope with greater patient numbers may be telemedicine: measures of visual function that can be self-administered at home or in the community, for the purposes of monitoring, screening, or patient prioritisation.

Several portable vision tests have been developed in the past decade, including measures of visual acuity (Bastawrous et al., 2015; Han et al., 2019; Chen et al., 2023), visual field loss (Ong et al., 2014; Vingrys et al., 2016; Jones et al., 2019; McLaughlin et al., 2024), and contrast sensitivity (Dorr et al., 2013; Kollbaum et al., 2014; Rodríguez-Vallejo et al., 2015; Habtamu et al., 2019; Elfadaly et al., 2020; Vivas-Mateos et al., 2020; Karampatakis et al., 2024). Often, however, such tests are evaluated in standardised clinical settings. The goal of the present study was to investigate the *resilience* of such tests to the potential confounding factors associated with an uncontrolled home environment, such as variations in lighting or seating type. Note that we use the new term “resilience” to refer to a test’s general robustness (in every measurable sense) to use in an uncontrolled home environment. A maximally resilient test would be one whose performance (*i.e.,* repeatability, duration, and ease of use) remained unchanged either when used at home unsupervised or in the clinic under supervision. In contrast, many conventional clinical measures, like letter charts and static perimeters (even those that may be relatively fast, accurate, and repeatable when used in clinic), would likely be relatively non-resilient (*i.e.,* would be slower, less accurate, and/or less repeatable at home), as they were never designed for use by patients unsupervised.

**Figure 1:**
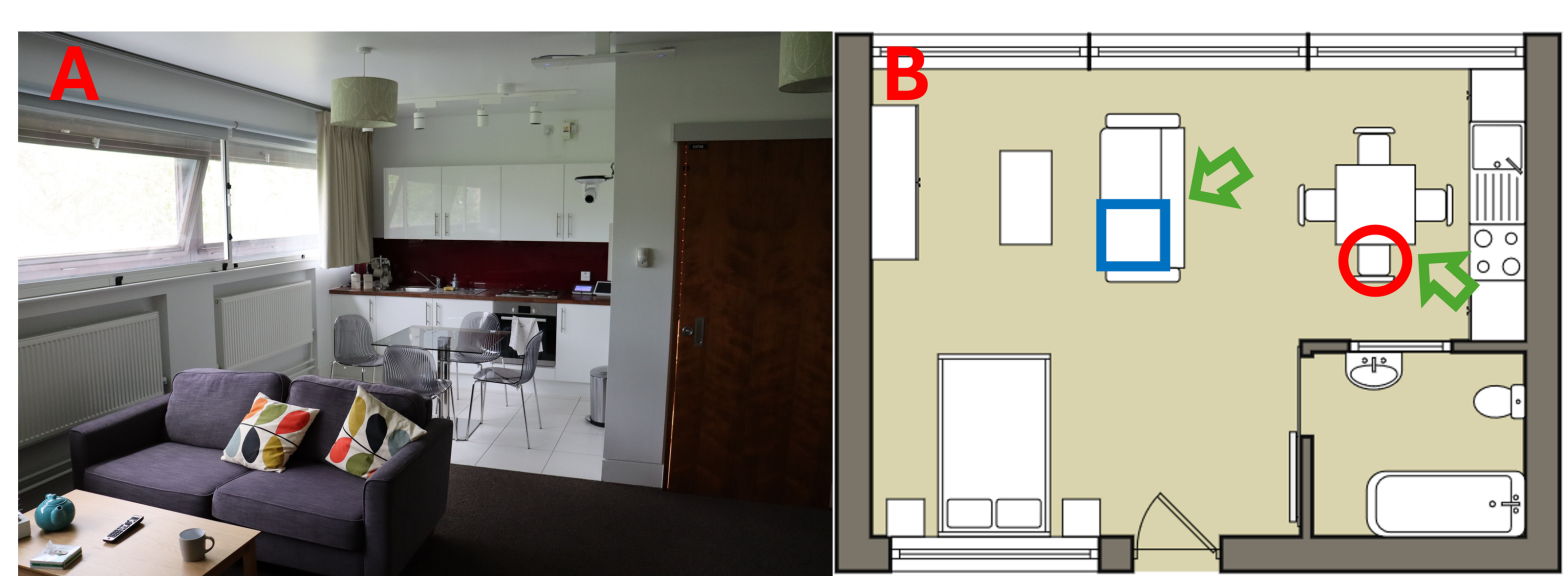
The home-testing environment. (A) Photo and (B) floor plan. Participants were randomly assigned to be seated on the sofa (blue square) or at the kitchen table (red circle). In a small number of cases a flood light was positioned at the locations of the green arrows, to further increase illumination and simulate strong glare. Large south facing windows extended across one wall, allowing almost constant sunlight within the hours of testing (08:30 –19:00).

To assess resilience, we asked a large cohort of normally sighted young adults to perform two self-administered contrast sensitivity (CS) tests in a specially constructed living room environment: a furnished apartment built within our university campus (see Fig. 1). A “control” cohort of normally sighted young adults was selected for this initial work in order to ensure an extremely homogenous sample (*e.g.*, in terms of in visual ability), allowing us to more easily detect any confounding effects from extraneous factors (between-subjects). In future, however, we plan to extend the present approach to patient groups (see *Discussion*). The use of the constructed apartment allowed us to conveniently let certain extraneous factors vary freely (*e.g.*, ambient illumination and time of day) while controlling others (*e.g.*, seating type) and allowed us to assess test performance within a relatively naturalistic setting.

In addition, in the present study we also investigated several other factors not directly related to the environment, but that might similarly limit the feasibility of home vision testing. First, we investigated task engagement. Some participants may be intrinsically more motivated or capable of performing vision tests, and such differences in motivation may be particularly pronounced when tests are self-administered in a home environment (*i.e.,* without a technician present to encourage or reassure the participant throughout the test). To investigate the effects of task engagement, we extracted response reliability metrics from visual field assessments performed as part of screening (visual field assessments being a notoriously challenging clinical procedure that produces a measurable quantity of “false positive” and “false negative” responses; Glen, Baker & Crabb, 2014) and examined whether participants who exhibited better reliability metrics subsequently produced higher or more consistent CS scores in the home environment. In addition, we also asked participants to answer several questions about the tests afterwards to examine whether people who rated the test more highly (*e.g.*, enjoyed it more) also produced better or more consistent CS scores. Second, we evaluated whether the level of instruction was important, as it may be that the performance of a home test depends on how carefully the procedure is explained to the patient. To this end, half of the participants had the test explained by a qualified optometrist, and half by a junior, non-clinical researcher. We hypothesised that participants who received the optometrist instructions might perform the tests more quickly or reliably. Third and finally, for the tablet-based test we furthered assessed screen cleanliness, as with home testing smudges may accumulate naturally over time through sustained use, and we cannot assume that patients will always remember to clean the screen. To this end, a subset of follow-up patients asked to repeat the tablet-based test with and without the presence of finger smudges.

The goal of the study is to assess whether these two CS home-monitoring tests are resilient to the extraneous factors that varied within our testing studio apartment. To do so, we assessed the impact of the illumination, seating type, time of day, participant motivation and level of instruction on the raw score, the repeatability and the duration of the two CS tests.

## Materials & Methods

### Participants

Participants were 107 normally sighted adults aged 18 to 30 years (median {interquartile range}: 20 {4} years). An overview of the demographics of the participants can be found in Table 1. While there was a clear imbalance in sex, this did not affect performance (see Table 2). As described in the introduction, we chose young, normally-sighted adults in order to minimise individual variability due to age or pathology (*i.e.,* to get a “pure” measure of the effects from extraneous factors alone). In order to provide a wider context, however, comparisons to data collected previously from cataract patients are also reported in Supplementary Section II.2.

**Table 1:** Demographic overview of the participants.

	Female	Male	Total
Number (*n* [%])	86 (80%)	21 (20%)	107
Age (median [IQR]; *years*)	20 (19 to 23)	22 (19 to 27)	20 (19 to 23)
Refractive error –spherical equivalent (median [IQR]; *dioptre*)	0 (−2.50 to 0.00)	0 (−3.25 to 0.00)	0 (−2.50 to 0.00)
Myopic (*n* [%])	41 (48%)	8 (38%)	49 (46%)
Hyperopic (*n* [%])	4 (5%)	2 (10%)	6 (6%)
Emmetropic (*n* [%])	41 (48%)	11 (52%)	52 (49%)
Visual Acuity (median [IQR]; L*ogMAR*)	0 (−0.04 to 0.06)	0 (−0.10 to 0.00)	0 (−0.07 to 0.06)

**Table 2:** Effect of binary variables on the test performance. Effect of various binary variables (participant sex, seating type, presence of a clinician) on test performance (CS score, repeatability, duration).

		SpotChecks			PopCSF		
		Raw score, (SD), *logCS*	Repeatability (SD) (—test 2–test 1—), *logCS*	Duration (SD), *secs*	Raw score (SD), *AUCSF*	Repeatability (SD) (—test 2–test 1—), *AUCSF*	Duration (SD), *secs*
Sex	Male (*n*= 21)	1.85 (0.07)	0.05 (0.04)	193 (49)	1.48 (0.18)	0.10 (0.09)	132 (20)
	Female (*n*= 86)	1.84 (0.14)	0.05 (0.05)	177 (46)	1.53 (0.15)	0.10 (0.08)	131 (21)
		*P* = 0.619	*P* = 0.534	*P* = 0.187	*P* = 0.236	*P* = 0.926	*P* = 0.923
Seating type	Sofa	1.85 (0.11)	0.06 (0.05)	185 (53)	1.52 (0.15)	0.11 (0.08)	135 (23)
	Table	1.83 (0.15)	0.05 (0.05)	176 (40)	1.52 (0.16)	0.10 (0.08)	128 (18)
		*P* = 0.543	*P* = 0.264	*P* = 0.315	*P* = 0.901	*P* = 0.451	*P* = 0.094
Administered	Clinician	1.85 (0.07)	0.06 (0.04)	170 (52)	1.51 (0.16)	0.12 (0.09)	134 (25)
by	Non-clinician	1.83 (0.17)	0.05 (0.05)	191 (39)	1.53 (0.15)	0.09 (0.08)	128 (15)
		*P* = 0.327	*P* = 0.107	*P* = 0.023	*P* = 0.458	*P* = 0.084	*P* = 0.158
Total		1.84 (0.13)	0.05 (0.05)	180 (47)	1.52 (0.15)	0.10 (0.08)	131 (21)
(All Participants)							

Normal vision was defined as no self-reported history of visual impairments or ocular disease, a habitually corrected letter acuity ≤ 0.2 logMAR in the better eye (ETDRS chart at three m; Precision Vision, Woodstock, Illinois, USA), a contrast sensitivity ≥ 1.5 logCS in the better eye (Pelli-Robson chart at one metre; Precision Vision, Woodstock, Illinois, USA), and a visual field of “within normal limits” or “borderline” in the better eye on a 24-2 visual field test on a Humphrey Visual Field (HVF) analyser using the SITA-FAST algorithm (Zeiss, Dublin, CA, USA). Two additional prospective participants failed screening (*i.e.,* *n* = 109 screened). Of these, one was “outside normal limits” in their visual field, while the other did not bring their prescription spectacles and so failed the acuity criterion.

Participants were recruited *via* adverts around the campus of City St George’s, University of London, and received £20 compensation for their time. Ethical approval was granted by the Optometry Proportionate Review Committee at City St George’s (#ETH2021-2265, #ETH2122-1956 and #ETH2324-0149). The study was carried out in accordance with the Declaration of Helsinki and participants provided informed written consent.

### Protocol overview

Participants performed two CS tests in a room furnished as an ordinary apartment (see Fig. 1).

We chose to measure CS as it is known to be symptomatic of a range of eye diseases (Pelli & Bex, 2013) such as glaucoma (Richman et al., 2010), macular degeneration (Kleiner et al., 1988), diabetic retinopathy (Stavrou & Wood, 2003), and cataract (Shandiz et al., 2011), and since, unlike visual acuity, it is relatively tolerant to variations in viewing distance (*i.e.,* likely making it a more pragmatic choice for home monitoring).

The two specific CS tests selected were the tablet-based PopCSF (Irida Health Ltd, London, UK) and pen-and-paper SpotChecks test (Precision Vision, Woodstock, Illinois, USA). Details of each are given below. Two different presentation modalities were chosen to evaluate their relative strengths and weaknesses (*e.g.*, we predicted that the pen-and-paper test would work better under strong illumination, and the digital test under low illumination). A third CS assessment, the Pelli-Robson, was also performed as part of initial screening, and was used as an approximate reference measure (though all three CS measures use different stimuli and would not be expected to perfectly correlate). The Pelli-Robson chart was chosen for pragmatic reasons as it is quick to perform and provides an outcome measure similar (in principle) to SpotChecks. In retrospect it would also have been desirable to use the CSV-1000 (VectorVision, Greenville, Ohio, USA) as an additional reference measure since, like PopCSF, it assessed the whole CSF.

Of the two tests used in the main study (PopCSF, SpotChecks), the starting test was randomly counterbalanced, and each test was performed twice (AABB/BBAA) in order to assess test–retest repeatability. All tests were performed using habitual refractive correction, and in the better eye only (fellow eye patched), though typically the difference between the visual acuity of the better and worse eye was minimal; Mean ΔlogMAR = 0.06 (standard deviation [SD]: 0.09). The better eye was determined by visual acuity (*n* = 83), by the dominant eye in case of a tie (*n* = 22), or defaulted to the right eye if dominance was unclear (*n* = 2).

Participants were consented and instructed by one of two randomly selected experimenters; one of whom was a first-year PhD student at the time of this study and the other a qualified clinical optometrist.

#### Creating a realistic home-testing environment

Participants were tested in a furnished studio apartment, constructed within the second floor of City St George’s, University of London. This space simulates a home environment and is also currently used, *e.g.*, by nursing students to practice ‘at-home’ interactions. As shown in Fig. 1, the apartment contained a bed, sofa, kitchen area with a kitchen table, and a bathroom. Participants were randomly directed to one of two seating types: the sofa (a more laid back or “relaxed” position) or at the kitchen table (a more upright or “active” position). However, participants were not instructed to sit in a specific manner (in keeping with a general intention to allow participants to behave in a naturalistic, ordinary way).

For most participants (*n* = 93), the blinds in the apartment were kept open and illumination was allowed to vary freely with time and the weather. Illumination was recorded using a lux meter (Aoputtriver AP-881D; Zhuhai, Guangdong, China) and is reported in the results as an independent variable. To further extend the range of illuminations, either the blinds were closed to achieve extremely low illumination (*n* = 3), or a flood light (Lepower ZSTGD-100W; Lepower Tec, Shenzhen, China) was used to provide very high illumination (*n* = 12).

Participants were given brief standardised verbal instructions on how to perform the test (*see below*). During the test, however, participants were free to do the test how they preferred without interference. While the experimenter was present in the room for the study, they stood out of the participants’ line of sight (usually on the sofa if the participant was seated in the kitchen and *vice versa*) and did not intervene or try to correct the participant, as per a “normal” home environment.

### The SpotChecks (pen and paper) contrast sensitivity test

**Figure 2:**
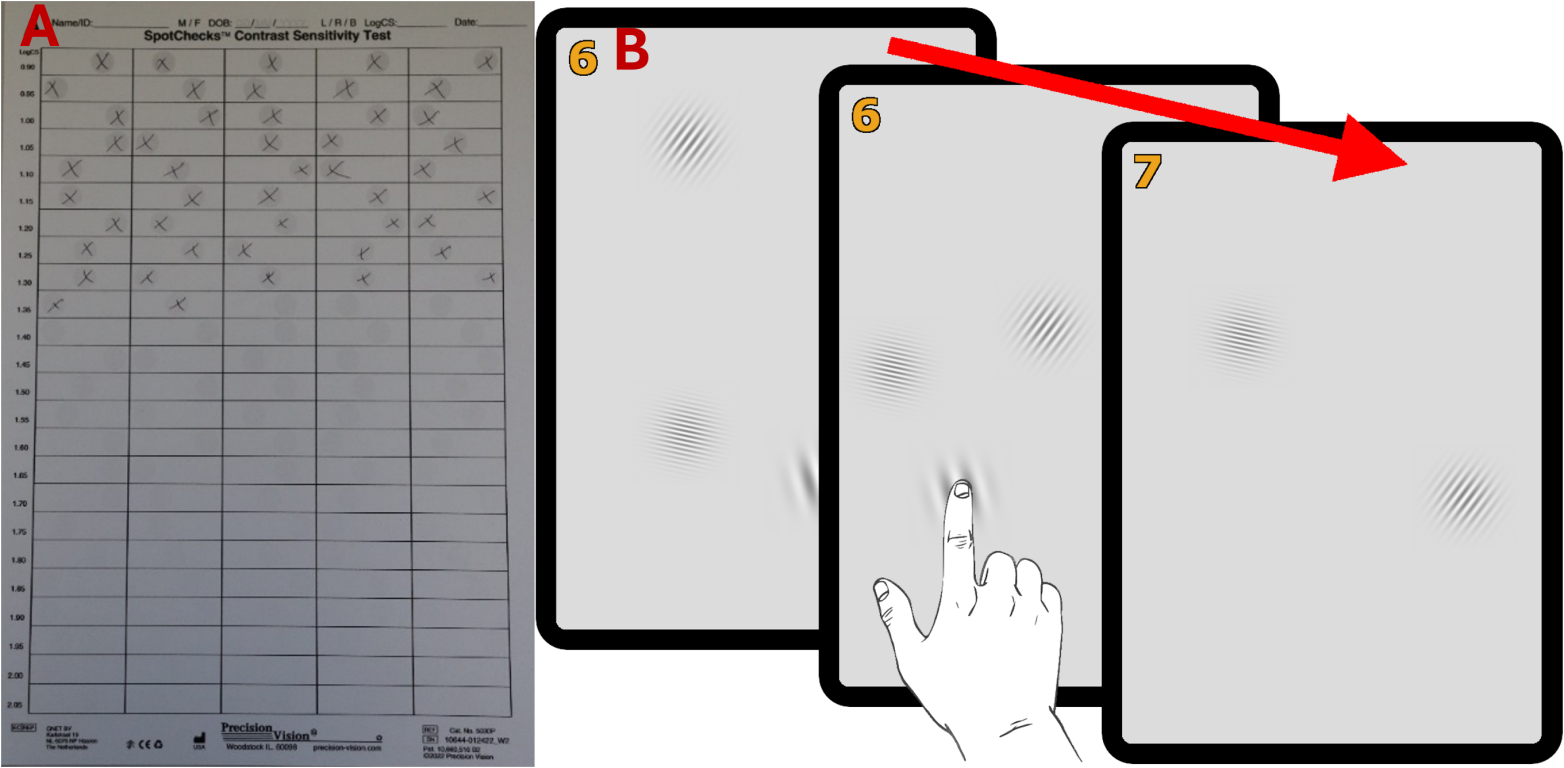
The contrast sensitivity tests. (A) A partially completed pen-and-paper SpotChecks sheet (visible stimuli marked in pencil with a cross). (B) Drawing of PopCSF mid-test. The leftmost tablet shows the initial version, with three different Gabors visible. The middle tablet shows the person tapping the screen at the location of one of the Gabors. The rightmost tablet shows the test after one of the Gabors was successfully “popped”, two Gabors now remain (but more will be periodically generated throughout the test). The number in the top left of the tablet shows how many Gabors were correctly “popped” by the participants, though this score was for motivational purposes only. The Gabors have been made easier to identify in this visual representation and are therefore not fully isoluminant as in the real test.

Details of SpotChecks have been described previously (Anderson, Mathew & Cheng, 2023; Bianchi et al., 2024; Crossland et al., 2024). In brief, the test consists of a single sheet of A4 paper, containing grey circles of varying contrast that the user must circle with a pen (see Fig. 2A). Contrast progressively decreases down the page (0.05 logCS per line), and participants were asked to mark each circle that they could see until they could not see another grey circle anymore. The output measure is an overall estimate of CS (-log10(Weber contrast [%]) of the smallest detectable contrast; in logCS).

Participants were free to take as much time as they wanted to complete the test. The participants were presented with the sheet of paper laid down on the table when seated at the table or handed to them when seated on the sofa. But they could hold or move the paper as desired. When seated on the sofa, some participants performed the test on their lap, while some used the coffee table. Others opted to place a book from the coffee table behind the test for stability. Some participants held the test closer or further away during the test, sometimes even holding up to the light to get a better look.

### The PopCSF (tablet based) contrast sensitivity test

As shown in Fig. 2B PopCSF (Elfadaly et al., 2020; Crossland et al., 2024; Michaelides et al., 2025) is a tablet-based contrast sensitivity test where the user is required to “pop” bubbles (horizontal Gabors of variable contrast and spatial frequency) by touching them as they drift across an isoluminant grey background. The test is designed to be game-like (*e.g.*, includes audiovisual feedback), and the exact location of each Gabor on the retina is not controlled though generally fall within the macula. For the exact specifications of the test, please consult the initial publication on PopCSF (see Elfadaly et al., 2020).

The test ran on an iPad Pro 11-inch (2nd Gen) tablet (Apple, Cupertino, CA, USA), with a 2,388 × 1,668-pixel touchscreen display (264 pixels per inch). Real time head tracking (*via* the integrated near-infrared TrueDepth camera) was used to modify the spatial frequency of the Gabors to adjust for the viewing distance. The TrueDepth camera had a precision of approximately one millimetre (Andrews et al., 2023) and a measurement error of approximately 5% of the target distance (Breitbarth et al., 2019). The screen was calibrated (linearised) using central measurements of screen luminance, made using a Konica Minolta LS100 luminance meter (Konica Minolta, Tokyo, Japan).

PopCSF measures the CS at various spatial frequencies and fits a 3-parameter contrast sensitivity function (Elfadaly et al., 2020). Here, the Contrast Sensitivity Functions (CSF) is summarised/reduced to a single scalar value by computing the area under the CSF (AUCSF) (Dorr et al., 2017).

### Analysis

Some data was lost. For seven participants (7%) data for PopCSF were not saved due to a technical error, while for two participants (2%) ambient illumination information was not recorded due to human error (see Supplementary Section I.I). Since the proportion of missing data was small, and since instances were independently randomly distributed, we do not believe these missing data materially affected the study conclusions.

Analyses were performed using R v4.4.1 (R Core Team, 2024). Correlations were calculated using Pearson correlation, while differences between conditions were evaluated using *t*-tests unless otherwise specified. Since six analyses were performed for each subsection of the results, a simple Bonferroni correction was applied. As a result, only *P* values <0.0083 were considered statistically significant. However, for completeness we also highlight where the uncorrected result is significant. This is only relevant for two findings (the duration of SpotChecks depending on the clinician experience and the repeatability of SpotChecks depending on the time of day), the potential consequences of this are discussed.

## Results

### Preliminary overview of results

Table 2 shows the overall effect of various binary variables (participant sex, seating type, and instructor’s clinical experience) on (1) contrast sensitivity scores; (2) test-retest repeatability, and (3) duration. In brief, this table shows that these three binary values do not have an impact on the score, repeatability or duration. However, specific results are described in the sections below (seating type in ‘Resilience to seating type’ instructor’s clinical experience in ‘ Resilience to level of instruction’).

Table 3 shows the overall effect of various continuous variables (illumination, time of day, participant feedback and clinical reliability (from perimetry)) on the same three dependent variables (raw scores, repeatability, duration). Again, these show in general no significant effects but are explored in more depth in the sections below (illumination in ‘Resilience to ambient illumination’, time of day in ‘Resilience to time of day’, participant feedback in ‘Participant feedback’ and clinical reliability in ‘HVF reliability score’).

**Table 3:** Effect of continuous variables on test performance. Effect of various continuous variables (illumination, time of day, participant feedback, clinical reliability) on test performance (CS score, repeatability, duration). Repeatability here is defined as the absolute difference between the first and second test, as the coefficient of repeatability cannot be used for a correlation.

		SpotChecks			PopCSF		
		Raw score, *logCS*	Repeatability (—test 2–test 1—), *logCS*	Duration, *secs*	Raw score, *AUCSF*	Repeatability (—test 2–test 1—), *AUCSF*	Duration, *secs*
Illumination	Pearson’s *r*	0.19	0.08	0.10	0.11	−0.11	0.16
	95% CI	−0.01 to 0.37	−0.11 to 0.27	−0.09 to 0.29	−0.09 to 0.29	−0.30 to 0.09	−0.04 to 0.35
		*P* = 0.058	*P* = 0.401	*P* = 0.294	*P* = 0.296	*P* = 0.274	*P* = 0.114
Time of day	Pearson’s *r*	0.02	−0.21	0.10	0.12	−0.07	0.02
	95% CI	−0.17 to 0.21	−0.38 to −0.02	−0.09 to 0.28	−0.08 to 0.31	−0.27 to 0.12	−0.18 to 0.21
		*P* = 0.836	*P* = 0.032	*P* = 0.319	*P* = 0.251	*P* = 0.464	*P* = 0.881
Feedback	Pearson’s *r*	0.10	−0.04	−0.16	0.05	0.07	−0.06
metric	95% CI	−0.09 to 0.29	−0.23 to 0.15	−0.34 to 0.03	−0.15 to 0.24	−0.13 to 0.26	−0.25 to 0.14
		*P* = 0.301	*P* = 0.655	*P* = 0.096	*P* = 0.642	*P* = 0.520	*P* = 0.582
HVF	Pearson’s *r*	−0.01	−0.12	−0.06	−0.18	0.04	−0.00
reliability	95% CI	−0.20 to 0.18	−0.30 to 0.08	−0.25 to 0.13	−0.37 to 0.01	−0.16 to 0.24	−0.20 to 0.19
metric		*P* = 0.896	*P* = 0.235	*P* = 0.512	*P* = 0.066	*P* = 0.689	*P* = 0.977

### Resilience to ambient illumination

Illumination varied from 0.09 (scotopic) to 2649 lux (high photopic) between participants. For context, 50–750 lux is the range typically recommended for indoor office environments (HSE, 2025).

**Figure 3:**
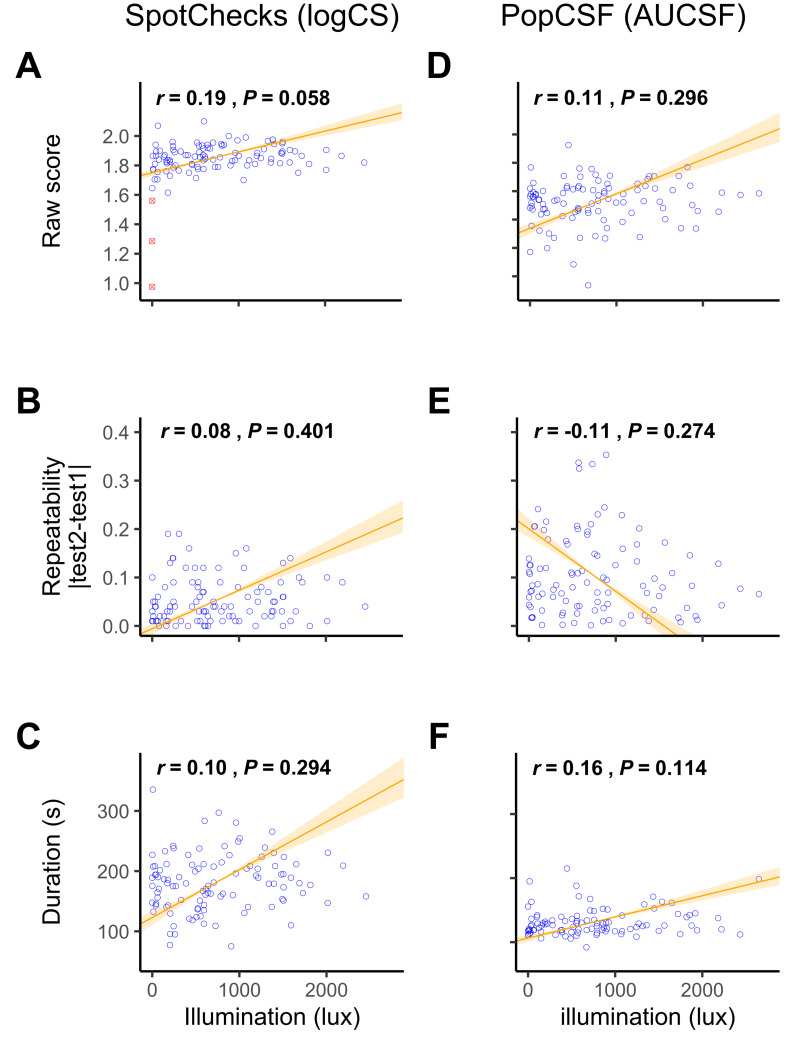
Resilience to Illumination. Scatterplot of the raw SpotChecks score (A), note that three participants (tested at ≤1 lux; shown as a red crossed circle) were removed as outliers, and the raw PopCSF score (D) with on the *x* axis the illumination in the room at the time of testing. Scatterplot of the absolute difference between the two SpotChecks scores (B) and the absolute difference between the two PopCSF scores (E) with on the *x* axis the illumination in the room at the time of testing. Scatterplot of the duration of SpotChecks (C) and the duration of PopCSF (F) with on the *x* axis the illumination in the room at the time of testing. Numerical values indicate Pearson correlation coefficients and significance values. The line signifies the standard major axis regression, with shaded regions indicating the slope’s 95% confidence interval.

For SpotChecks (Figs. 3A–3C), there was no correlation between illumination and test-retest repeatability (*r*_103_ = 0.08, *P* = 0.401), or between illumination and test duration (*r*_103_ = 0.10, *P* = 0.294). There was a weak positive correlation between illumination and raw CS score (*r*_103_ = 0.27, *P* = 0.006), indicating that for SpotChecks, CS scores improved with illuminance. However, if the tests conducted at ≤1lux (scotopic) were removed, this correlation ceases to be significant (*r*_100_ = 0.19, *P* = 0.058). Given that for those tested at ≤1 lux it was so dark that participants could barely see the sheet of paper, we believe that no reasonable person would ever attempt the test in such conditions. This indicates that ambient lighting is not a practical concern for SpotChecks. We also conducted a formal piecewise linear analysis which indicated that anything above 11 lux did not substantively affect CS score on SpotChecks (see Supplementary Section II.3 and Fig. 3).

For PopCSF (Figs. 3D–3F) there was no correlation between illumination and score (*r*_98_ = 0.11, *P* = 0.296), repeatability (*r*_98_ = −0.11, *P* = 0.274), or test duration (*r*_97_ = 0.16, *P* = 0.114).

Overall, these analyses indicate that variations in indoor illumination levels have no substantial effect on test performance for either test, except in extremely dark cases for the pen-and-paper test.

### Resilience to seating type

Next, we investigated whether performing the test sitting in a more relaxed posture (sitting on a sofa) or a more active posture (sitting at a table) could alter test performance.

**Figure 4:**
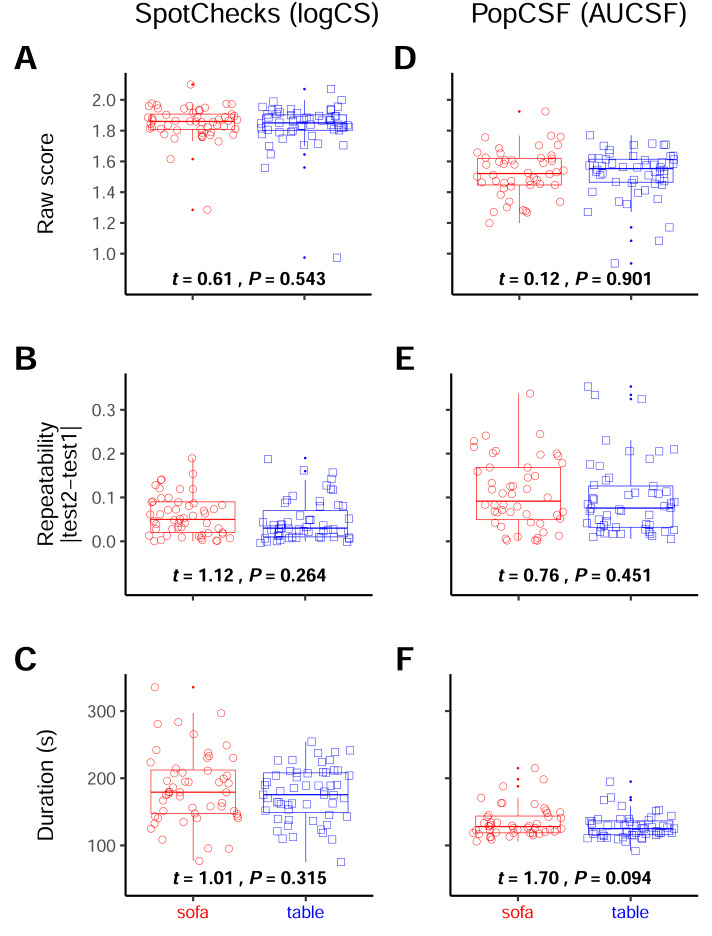
Resilience to seating type. Boxplots of the raw score (A & D), the repeatability (B & E) and the duration (C & F) separated by the participants that performed the test at the table, and those that performed them on the sofa. For SpotChecks the raw score and repeatability are in LogCS and for PopCSF those are in AUCSF, both duration graphs are in seconds. The numbers represent the *t*-test statistics and significance values.

For SpotChecks (Figs. 4A–4C) there was no effect of seating position (sofa *vs.* table) on score (*t*_101_ = 0.61; *P* = 0.543), repeatability (*t*_105_ = 1.12; *P* = 0.264), or duration (*t*_94_ = 1.01; *P* = 0.315).

Likewise, for PopCSF (Figs. 4D–4F) there was no effect of seating position on score (*t*_98_ = 0.12, *P* = 0.901), repeatability (*t*_98_ = 0.76, *P* = 0.451), or duration (*t*_87_ = 1.70, *P* = 0.094).

Overall, these analyses indicate that seating type has no substantial effect on test performance, either for the pen-and-paper SpotChecks or the tablet-based PopCSF.

### Resilience to time of day

**Figure 5:**
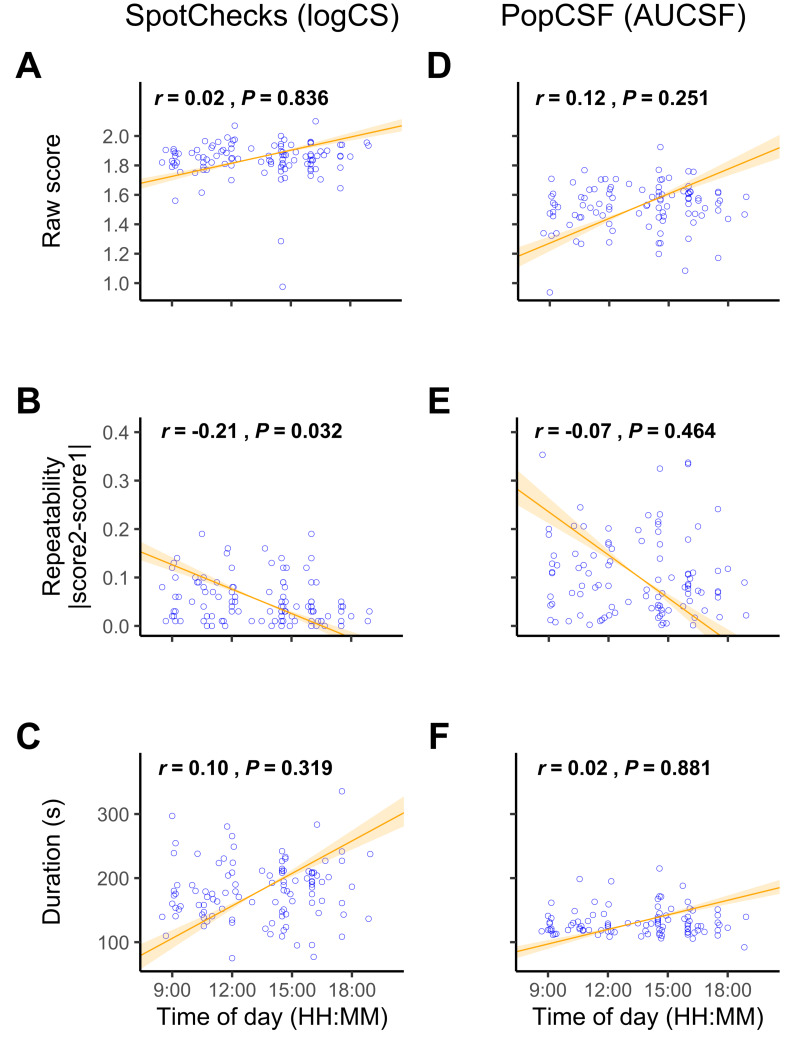
Resilience to time of day. Scatterplot showing dependent variables for (A–C) SpotChecks and (D–F) PopCSF as a function of the time of day. Numerical values indicate Pearson correlation coefficients and significance values. The line signifies the standard major axis regression, with shaded regions indicating the slope’s 95% confidence interval.

For Spotchecks (Figs. 5A–5B) there was no correlation between the time of day and score (*r*_105_ = 0.02, *P* = 0.836) or between time of day and test duration (*r*_105_ = 0.10, *P* = 0.319). As shown in Fig. 5C, there was a weak negative correlation between time of day and the precision (repeatability) of SpotChecks (*r*_105_ = −0.21, *P* = 0.032), but this ceased to be significant following Bonferroni correction (*P* < 0.008).

For PopCSF (Figs. 5D–5F) there was no correlation between the time of day and score (*r*_98_ = 0.12, *P* = 0.251), repeatability (*r*_98_ = −0.07, *P* = 0.464), or test duration (*r*_97_ = 0.02, *P* = 0.881).

Overall, these analyses indicate that the time of day has no significant impact on test performance.

### Resilience to participant motivation

Some participants may be inherently more invested in performing well on a test, and these differences in task motivation may manifest as differences in performance. This is always a concern for any test, but we predicted the effects might be particularly acute/pronounced in a telemedicine environment, with no experimenter to actively monitor/encourage the participant. We used two proxies for motivation: (1) whether the participant rated the test positively; (2) and whether the participant was consistent during the HVF test.

#### Participant feedback

Participants were asked five feedback questions after the experiment: whether they (i) understood the test, and found it (ii) easy, (iii) enjoyable, (iv) hard to concentrate on, and (v) tiring. Participants would rate these questions using a five-point Likert scale from (1) strongly disagree to (5) strongly agree. Their answers to these five questions were combined into one ‘feedback metric’ which ranged from 5 to 25, with 5 meaning that the participant was extremely negative on each subject and 25 meant that they were extremely positive about the test.

For SpotChecks (Fig. S4A) there was no significant relationship between the feedback metric and contrast score (*r*_102_ = 0.10, *P* = 0.301), but only when excluding the 3 participants tested at ≤1 lux. If included, there was a significant relationship (*r*_105_ = 0.26, *P* = 0.006), as these 3 individuals scored extremely badly on the test and rated the test very negatively. Even with these participants included, there was no correlation between the feedback score and the repeatability (*r*_105_ = −0.04, *P* = 0.655) or test duration (*r*_105_ = −0.16, *P* = 0.096) (Figs. S4B–S4C).

For PopCSF (Figs. S4D–S4F) there was no correlation between the feedback metric and CS score (*r*_98_ = 0.05, *P* = 0.642), repeatability (*r*_98_ = 0.07, *P* = 0.520), or test duration (*r*_97_ = −0.06, *P* = 0.582).

#### HVF reliability score

The HVF provides three variables to indicate the reliability of a visual field report, the percentage of false positives, false negatives and fixation losses. These indicate how consistent a participant is during a visual field test and have been shown to affect HVF test performance (Lee, Zulauf & Caprioli, 1994). These three percentages were summed into a composite score that shows how consistent a participant was, labelled here as the “HVF reliability metric”.

For SpotChecks (Figs. S5A–S5C) there was no correlation between the HVF reliability metric and the score (*r*_105_ = −0.01, *P* = 0.896), repeatability (*r*_105_ = −0.12, *P* = 0.235), or test duration (*r*_105_ = −0.06, *P* = 0.512).

For PopCSF (Figs. S5D–S5F) there was no correlation between the HVF reliability metric and the score (*r*_98_ = −0.18, *P* = 0.066), repeatability (*r*_98_ = 0.04, *P* = 0.689), or test duration (*r*_97_ = −0.00, *P* = 0.977).

Overall, these analyses indicate that participant motivation has no substantial effect on test performance.

### Resilience to level of instruction

Participants largely “tested themselves”. However, as per real clinical practice somebody had to explain what to do beforehand. For *n* = 53 participants, this explanation was given by author PFR (who at the time of the study was a first year PhD student), while for the other *n* = 54 participants, this explanation was given by author MR: qualified clinical optometrist with formal training in explaining vision testing procedures.

**Figure 6:**
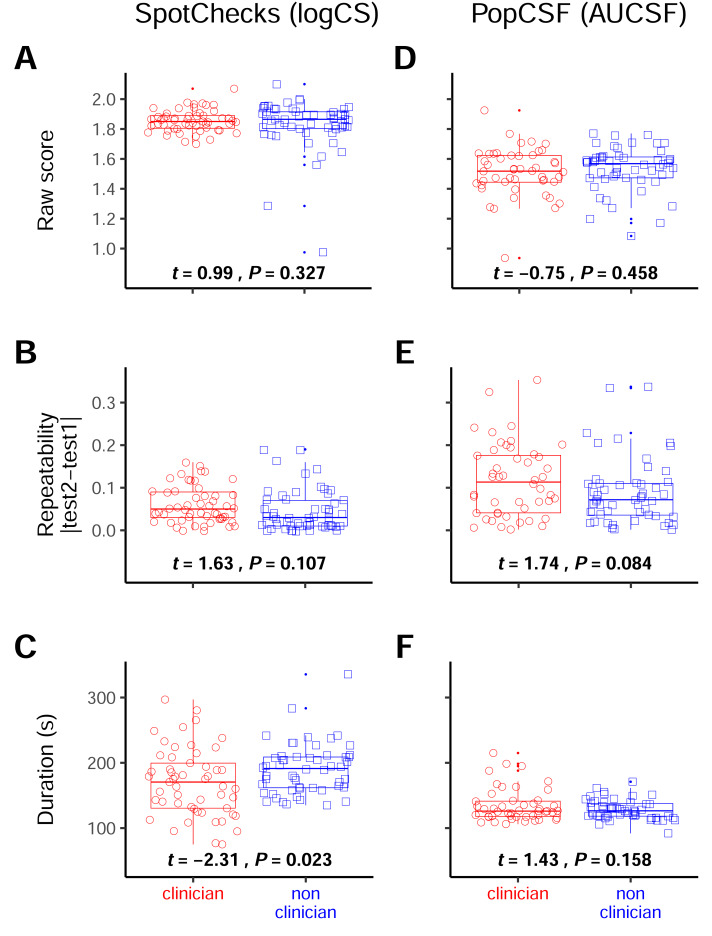
Resilience to the presence of a clinician. Boxplots of the raw score (A & D), the repeatability (B & E) and the duration (C & F) separated by the participants that were tested by the clinician and those that were tested by the non-clinician. For SpotChecks the raw score and repeatability are in LogCS and for PopCSF those are in AUCSF, both duration graphs are in seconds. The numbers represent the *t*-test statistics and significance values.

For SpotChecks (Figs. 6A–6B) there was no effect of the experimenter’s clinical experience on the score (*t*_72_ = 0.99, *P* = 0.327) or repeatability (*t*_104_ = 1.63, *P* = 0.107). There was, however, a significant difference between the participants tested by either experimenter on the duration (*t*_96_ = −2.31, *P* = 0.023), with the people completing the test on average 20 s (11%) faster when instructed by the clinician (see Fig. 6C). This was not significant after considering a Bonferroni corrected significance value, however (*P* <  0.008).

For PopCSF (Figs. 6D–6F) there was no effect of the experimenter on the score (*t*_96_ = −0.75, *P* = 0.458), repeatability (*t*_95_ = 1.74, *P* = 0.085) or test duration (*t*_76_ = 1.43, *P* = 0.158).

Overall, these analyses indicate that the performance of tests in a home type environment can be measurably affected by the person delivering the test instructions (even when the instructions themselves are standardised), but that the nature/magnitude of this effect is relatively slight (in this instance, a small difference in test duration).

### Resilience to screen smudges

**Figure 7:**
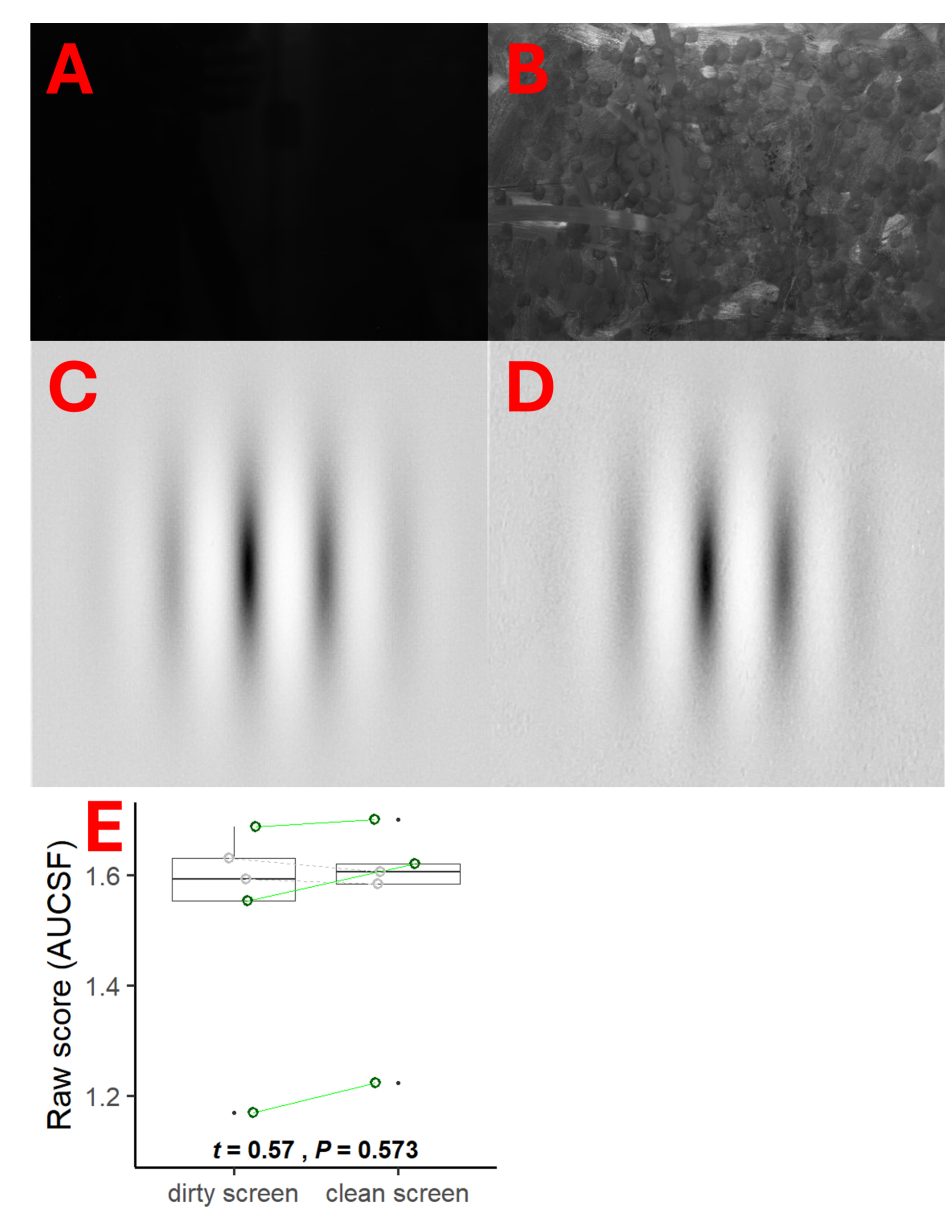
Resilience to screen smudges. Image of the iPad with the screen turned off and a clean screen (A), and that same image with a smudged screen (B). A zoomed image of the PopCSF screen showing a Gabor patch with a clean screen (C) and with a dirty screen (D). A boxplot (E) of the mean score (AUCSF) of each participant (coloured) and whether a clean screened caused them to perform better (green continuous line) or worse (grey striped line). A boxplot of the overall effect is also shown within the figure. Numerical values indicate the results of a *t*-test on the data.

For a pen-and-paper test such as SpotChecks a new, fresh sheet is used for every assessment. In contrast, a tablet-based test such as PopCSF is reusable. Over time, screen smudges are liable to occur, possibly obscuring low contrast stimuli. In our main experiment any effects of smudging were minimised as we thoroughly cleaned the screen between participants. In the real world, however, a single patient using the device at home may not be so diligent. To investigate the potential effect of screen smudging, we therefore performed a follow-up experiment in which *n* = 5 randomly selected participants were called back to perform an additional, intensive testing session comprised of 24 successive PopCSF tests. Participants performed groups of six tests starting randomly with a clean (see Fig. 7A) or dirty screen (see Fig. 7B) and alternating between the two conditions until they had completed 24 tests in total (12 clean, 12 dirty).

The screen was smudged by the participants themselves by greasing their hands with crisps and wiping it all over the screen, making sure that the entire screen was covered in grease. The difference between the clean and dirty screen was very clearly visible with the screen turned off (clean: Fig. 7A, dirty: Fig. 7B). The same pictures were made of the same clean and dirty screen displaying a Gabor as PopCSF would present it (clean: Fig. 7C, dirty: Fig. 7D). Visual inspection shows that there was a huge difference when the screen was turned off (Figs. 7A & 7B) but hardly any difference when the screen was displaying the Gabor (Figs. 7C & 7D). This was likely due to the light reflecting off the grease on the screen when the screen was turned off, but that effect was overpowered by the light emanating from the screen when it was turned on.

To try to quantify the effect of the smudging we used a simple pixel intensity analysis, where each pixel of the image was ranked from 0 (completely black) to 255 (completely white). For the images with the screen turned off (Figs. 7A & 7B), the mean intensity was 7 (SD: 1) for the clean screen and 80 (SD: 15) for the dirty screen. For the image of the Gabor (Figs. 7C & 7D) the mean intensity was 213 (SD: 19) for the clean screen and 212 (SD: 18) for the dirty screen. This shows that even though the smudginess is very clearly a factor when the screen is turned off, it hardly makes any difference when the screen is turned on.

As shown in Fig. 7E, two of the five participants actually performed slightly better on average (higher CS) with the smudged screen, and there was no significant difference in performance between the clean and dirty screen (*t*_115_ = 0.57, *P* = 0.573). This indicates that smudging the screen does not substantially affect the performance on the tablet-based test.

## Discussion

With the growing interest in vision home monitoring (Han & Jones, 2019; Che Hamzah, Daka & Azuara-Blanco, 2020) the present study aimed to evaluate the extent to which various extraneous factors (seating type, illumination, time of day, motivation, instructors clinical experience, and screen smudging on two portable CS) might confound two portable CS tests (one pen-and-paper based, one digital tabled-based).

Contrary to our expectations, none of these factors appeared to be substantive confounds, suggesting that these (and potentially other similar portable CS tests—see below) are relatively resilient to the extraneous factors examined in this study. Specifically, there were no significant effects of the seating type, motivation or presence of a clinician on the CS score, repeatability and duration of either test. And while there was a significant effect of the illumination on the pen-and-paper based test, SpotChecks, this was manifest only in extremely dark conditions (<11 lux) in which no reasonable person would ever attempt to perform the test.

For SpotChecks there were two borderline effects that ceased to be statistically significant following Bonferroni correction. There was a 20 s (12%) increase in time spent on the test when tested by a non-clinician instead of a clinician and a weak correlation (*r* =  − 0.21) between the repeatability and the time of day. These may warrant further investigation in future, but even if they prove significant, the effect sizes are small (*i.e.,* they would unlikely affect the clinical utility or acceptability of the intervention).

One particularly novel and important finding of the present study was that there was no effect of the seating location on the resilience of either CS test. When seated at the table, participants would keep the test on the table but when seated on the sofa, they had no easy spot to put the test. This meant people were encouraged to improvise, *e.g.*, holding the PopCSF tablet on their lap or grabbing a book to put under the SpotChecks sheet in order to write on it. This is encouraging, as it suggests that vision home monitoring could potentially be performed without the need for strict instructions or unrealistic assumptions regarding patient compliance.

### Previous literature

Many previous studies using portable vision tests such as PopCSF or SpotChecks have used standardised testing environments (Elfadaly et al., 2020; Nixon & Flinn, 2021; Rijal, Cheng & Marsack, 2021; Anderson, Mathew & Cheng, 2023; Crossland et al., 2024). Two studies, however, had patients perform SpotChecks at home (Bianchi et al., 2024; Vu et al., 2024), and both reported similar results at home and in the clinic. This is consistent with our present findings, where we did not find any extraneous factors that substantially affect the resilience of the home CS tests.

A study by Abraham et al. (2023) investigated the effect of several colour filters and changes in illumination on participants with moderate to severe vision impairment. They found no changes in CS when tested at 100, 300, 700 or 1,000 lux. This is consistent with our findings, as we did not find an effect of illumination (ranging from 0.1 to 2,453 lux) on the SpotChecks score.

Similarly, looking beyond the particular CS measures employed in the present study, Cox, Norman & Norman (1999) investigated the effect of illumination on the performance of the Pelli-Robson CS chart. At 4 m they found that increasing illumination from 9 lux to 900 lux caused an increase in CS of roughly 1 logCS. However, at one metre distance, Cox and colleagues found no significant effect of illumination on CS. This again is broadly consistent with our present findings, since in the present study both SpotChecks and PopCSF were held at arm’s length (*i.e.,* <1 m).

Montolio et al. (2012) systematically investigated the factors that influence visual field performance in clinics and found that test outcome varied by the time of day that the participant was tested. This is inconsistent with our findings, as we found no effect of the time of day on the contrast sensitivity score as measured by either of our two home tests. However, a visual field test is a much more intense procedure than the CS tests featured in our study, which may explain why we found no effect of the time of day on the raw score of either test.

Vingrys et al. (2016) and Harris et al. (2022) investigated whether the outcome and reliability of the tablet-based visual field test (Melbourne Rapid Fields) was affected by illumination. Both studies found no effect of illumination on the outcome or reliability of the test, which is consistent with our present findings.

Finally, we can compare our repeatability findings to recent studies that tested PopCSF, SpotChecks either in clinic or at home. A recent study by Crossland et al. (2024) which studied both SpotChecks and PopCSF in the clinic reported a CoR_95_ of 0.13 logCS and 0.29 AUCSF respectively, similar to our recorded CoR_95_ of 0.14 logCS and 0.26 AUCSF respectively (see Supplementary Section II.1). Another study by Vu et al. (2024) calculated the inter-day repeatability of SpotChecks at 0.18 logCS in the clinic and 0.18 logCS at home, similar to our intra-session repeatability of 0.14 logCS. Our repeatability was likely lower as we tested in one session (intra-session) while in the study by Vu et al. the repeatability was assessed over multiple sessions (inter-day). This seems to indicate that there is no change in repeatability when testing at home, in the clinic, or in our uncontrolled simulated home environment.

### Limitations and future work

A key limitation of the present study is that we only recruited normally sighted young adults. This was intentional, as it allowed us to investigate the effect of the extraneous factors without large individual differences. However, long term it would be necessary to extend the present approach to a wider age range and in those with visual disorders. For instance. it is known that due to age-related changes throughout the eye older adults often have more trouble seeing in low light conditions (Owsley, 2011) and in the presence of glare (Kimlin, Black & Wood, 2017), meaning that, for example, home tests may be less resilient to changes in illumination in older adults. Our next step will there be to confirm the initial findings on the resilience of these tests in participants with a wider age range and in those with visual disorders. Additionally, since this was an exploratory study, we could not conduct a formal power analysis before starting the study. However, by recruiting over 100 participants we believe that we achieved a proper spread of participants over our variables to answer our research questions.

Second, future studies could also investigate additional extraneous factors not considered in the present study, such as the auditory distractions from music, tv, or people talking. It might also be instructive to study in the future the effects of inappropriate refractive correction. Thus, when unsupervised it is likely that some patients might forget to wear their spectacles or wear inappropriate ones (*e.g.*, distance rather than reading glasses) and this could also be an additional source of home-measurement error. While this would be most important for home visual acuity testing, it might also cause issues for CS testing. For example, in visual field testing (which is effectively a form of peripheral CS test), a 1 dioptre refractive error has been shown to decrease the sensitivity by 1.26 dB in the central 6 degrees of the visual field (Weinreb & Perlman, 1986). Finally, for screen-based tests there might also be concerns about screen calibration if patients are expected to download the test onto their own devices. Though it should be noted that the PopCSF test used in the present study is not widely available to the general public for that very reason, and potential solutions for “self-calibrating” (Han & Jones, 2019) or “psychophysically-calibrated” tests (To et al., 2013) have been suggested.

A third limitation of the present study is that we only investigated two contrast sensitivity (CS) tests. However, we have no reason to expect other similarly CS tests (*e.g.*, PeekCS (Habtamu et al., 2019) or qCSF (Dorr et al., 2013)) to perform markedly differently, and we predict similar levels of resilience. What is less clear, however, is how well the present findings would generalise to other measures of visual function (*e.g.*, visual acuity, colour vision, visual fields), which may be more or less susceptible to extraneous factors. Thus, while visual acuity has, for example, been shown to be measurable by patients at home (Crossland et al., 2022), it places more demands on users (*e.g.*, to maintain a precise and constant viewing distance), and so may potentially be less resilient to unsupervised use in a home setting.

Fourth, for practical reasons we used a standardised testing location rather than participants’ actual homes. The room was set up as closely as possible to an actual home and contained all the furniture and amenities of a real studio apartment. This provided an ideal location to perform this study, as we could let many extraneous variables vary freely (*e.g.*, illumination) while still having a semi-standardised environment (*e.g.*, windows in the same place). However, there is clearly a difference between being in a home with an experimenter present and being in your own home. For example, participants seated on the sofa where mostly sitting neatly and would not lie down on it as one might do at home. Additionally, participants were instructed on how to do the test right before performing it. Normally there would be a much longer period between the explanation and when the participant would perform the test at home. Therefore, it would be desirable to build upon these initial findings in the future by letting people test themselves fully unmonitored at home, though in such circumstances it would be more challenging to measure and/or account for extraneous factors such as illumination and seating position.

Fifth and finally, this study is only meant to assess the resilience of home monitoring vision tests, not the much wider question of whether patient home monitoring is desirable or effective. The latter further encompass issues such as value for money, clinical utility and patient acceptability that fall outside the scope of the present study. For instance, research by Dave et al. (2024) showed that while patients think home monitoring could be beneficial, it could also create non-trivial challenges surrounding issues such as patient anxiety, digital exclusion, and fatigue.

## Conclusions

Extraneous factors (seating type, time of day, presence of a clinician, and motivation) did not significantly affect the contrast sensitivity score, repeatability and duration of either a pen-and-paper or digital CS test.

The findings indicate that the portable CS tests may be relatively resilient to variations in environmental conditions and might therefore be appropriate for patient home monitoring. Next, these results will need to be extended to older individuals and those with vision deficits, as well as testing the individuals in their actual homes.

## Supplemental Information

10.7717/peerj.20657/supp-1 Supplemental Information 1 Supplemental tables and figures

10.7717/peerj.20657/supp-2 Supplemental Information 2 Main dataset

10.7717/peerj.20657/supp-3 Supplemental Information 3 Dataset of the smudging follow-up experiment

## Additional Information and Declarations

### Data Availability

The following information was supplied regarding data availability: The raw data are available in Supplementary Files.
