# Supplementary material for: Assessing the resilience of portable vision tests to an uncontrolled home environment
Source: PeerJ. 2026 Feb 20;14:e20657. doi: 10.7717/peerj.20657 (PMC12927602; doi:10.7717/peerj.20657)
Supplement: Supplemental Information 1 [file peerj-14-20657-s001.docx]

**SUPPLEMENTAL MATERIAL** for “*Assessing the resilience of portable vision tests* *to an uncontrolled home environment*”

**I. METHODS**

***I.1 Missing data***

109 participants were recruited and screened for this study, of which 107 passed our screening procedure.

For analyses involving PopCSF, seven participants were excluded (*n*=100 included). For 5 participants this was due to a technical error (a bug in the code that rendered the data unusable). An additional two participants were excluded, one because they did not press the screen a single time during the PopCSF test and one who did not wear their glasses for the PopCSF tests.

In addition, for analyses involving illumination levels, two participants were excluded as the illumination level was not recorded due to human error. While for analyses of test duration, one participant was excluded as the test duration was not recorded due to human error.

**II RESULTS**

***II.1 Repeatability of SpotChecks and PopCSF***

In the main text the test-retest variability (|test2 – test1|) were used to correlate those values with the investigated extraneous factors. However, to facilitate comparisons with previous literature using CoR_95_ we have added those values in this section. CoR_95_ values for each extraneous variable investigated (in the case of continuous variables the variable was split on the median) can be found in **Supplementary Table 1**. Additionally, Bland-Altman plots of both SpotChecks and PopCSF can be found in **Supplementary Figure 1**.

|  | SpotChecks | | PopCSF | |
| --- | --- | --- | --- | --- |
|  | Splitting factor | CoR_95_ Repeatability (CI_95_), *LogCS* | Splitting factor | CoR_95_ Repeatability (ci_95_), *AUCSF* |
| Illumination | < 606.5 *lux* (*n*=51) | 0.14 (0.11 – 0.18) | <641 lux (*n*=50) | 0.26 (0.21 – 0.31) |
| (lux) | > 606.5 *lux* (*n*=51) | 0.13 (0.11 – 0.15) | >641 lux (*n*=50) | 0.26 (0.20 – 0.31) |
|  |  |  |  |  |
| Seating Type | Sofa (*n*=51) | 0.15 (0.12 – 0.17) | Sofa (*n*=48) | 0.26 (0.22 – 0.31) |
|  | Table (*n*=53) | 0.13 (0.10 – 0.16) | Table (*n*=52) | 0.25 (0.20 – 0.31) |
|  |  |  |  |  |
| Time of Day | < 14:05 (*n*=52) | 0.15 (0.12 – 0.18) | <14:30 (*n*=49) | 0.26 (0.21 – 0.31) |
| (HH:MM) | > 14:05 (*n*=52) | 0.12 (0.09 – 0.15) | >14:30 (*n*=43) | 0.25 (0.19 – 0.32) |
|  |  |  |  |  |
| Feedback | < 22 (*n*=42) | 0.15 (0.12 – 0.19) | < 21 (*n*=47) | 0.24 (0.20 – 0.29) |
|  | > 22 (*n*=47) | 0.13 (0.10 – 0.16) | > 21 (*n*=42) | 0.27 (0.21 – 0.33) |
|  |  |  |  |  |
| HVF reliability | < 9.5 (*n*=52) | 0.14 (0.11 – 0.16) | < 10 (*n*=49) | 0.25 (0.19 – 0.31) |
| (*%*) | > 9.5 (*n*=52) | 0.14 (0.11 – 0.17) | > 10 (*n*=43) | 0.27 (0.22 – 0.32) |
|  |  |  |  |  |
| Administered by | clinician (*n*=53) | 0.14 (0.12 – 0.17) | clinician (*n*=48) | 0.28 (0.23 – 0.34) |
|  | non-clinician (*n*=51) | 0.13 (0.10 – 0.16) | non-clinician (*n*=52) | 0.23 (0.18 – 0.28) |
|  |  |  |  |  |
| Total | *n*=104 | 0.14 (0.12 – 0.16) | *n*=100 | 0.26 (0.22 – 0.29) |
|  |  |  |  |  |

**Supplementary Table 1:** **CoR_95_ values of each investigated extraneous variable for both SpotChecks and PopCSF.**


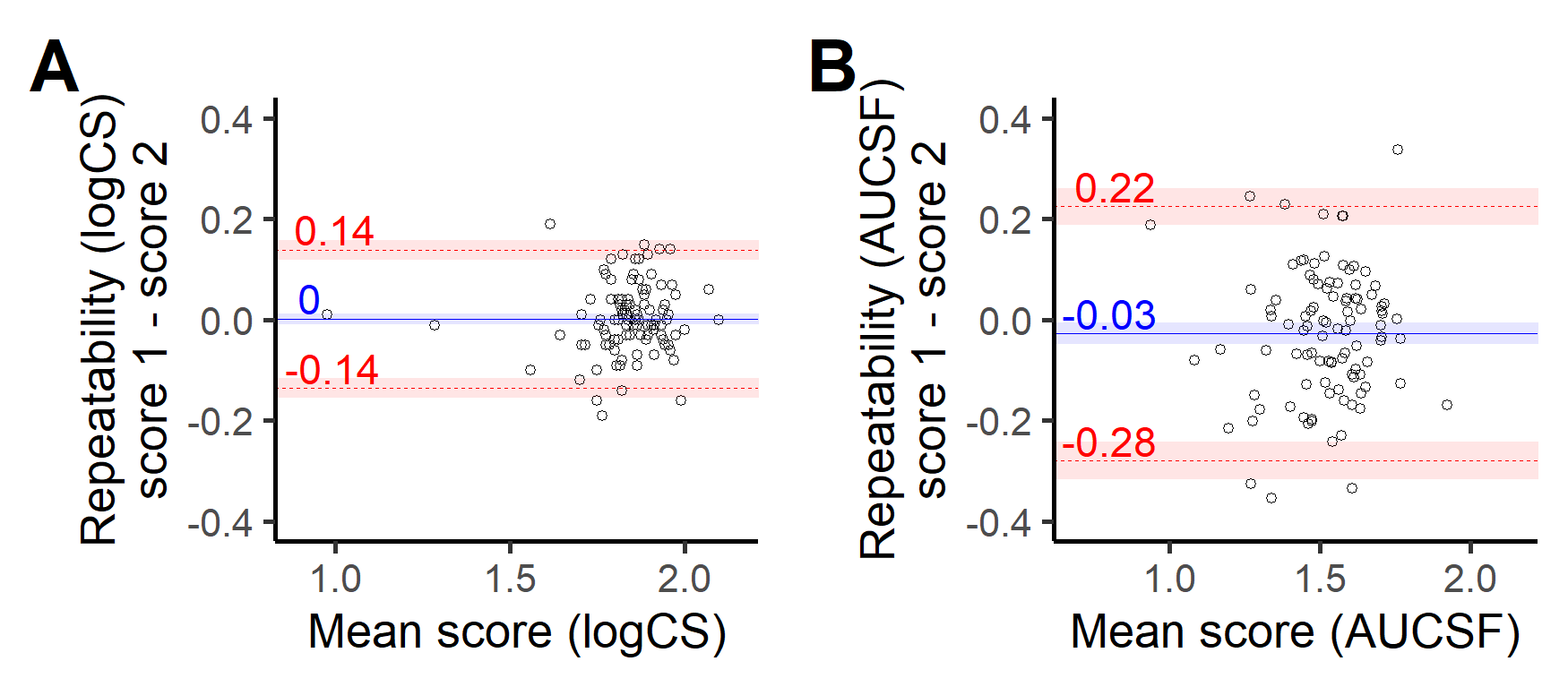


***Supplementary Figure 1: Repeatability plots of the CS tests.*** *Bland-Altmann plots of SpotChecks (****A****) and PopCSF (****B****). Blue continuous central line shows the mean difference, the red dashed lines the upper and lower limits of agreement. The numbers written close to the lines represent the values of the mean difference and limits of agreement. Shaded areas represent the 95% confidence intervals of the mean difference and limits of agreement.*

***II.2 Comparison to cataract patients***

Here we compare the data from the present data with previously collected data in older cataract patients.^26^ As shown in **Supplementary Figure 2**, The CS scores were considerably lower and more varied in cataract patients versus the healthy young adults in the present study, as one would expect.

~~
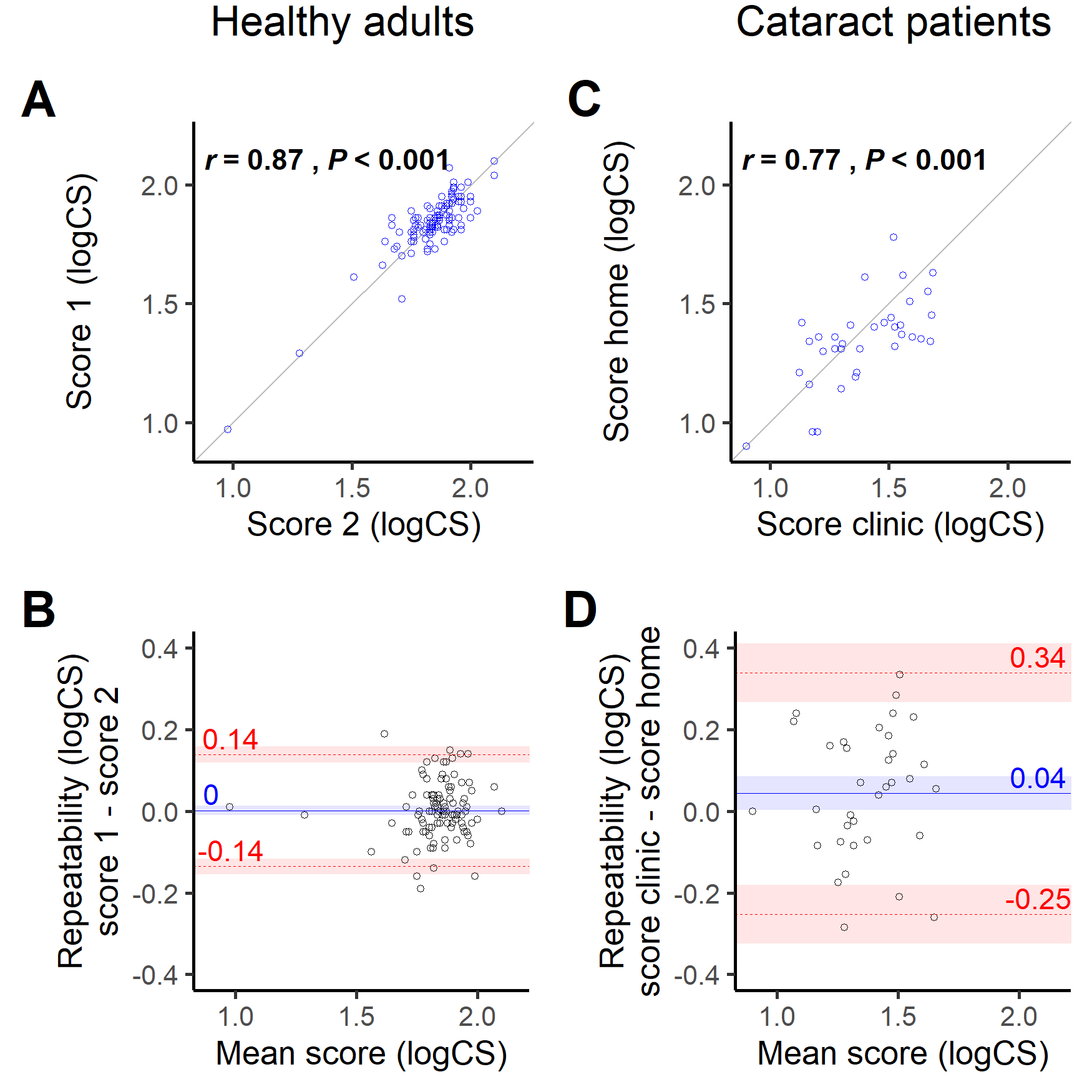
~~

***Supplementary Figure 2: Figures comparing both tests to the reference standard.*** *Scatterplots (****A****&****C****) and Bland-Altman plots (****B****&****D****) comparing the repeatability of SpotChecks done in young adults twice in the simulated apartment (****A****&****B****) and in cataract patients at home and in the clinic (****C****&****D****). The patient data was retrieved from the study by Bianchi et al.*^26^

**II.3: Resilience to ambient illumination**

To determine exactly what the minimum illuminance is required to perform the SpotChecks, we refitted the data in **Figure 3A** using piecewise linear regression. A linear regression on the SpotChecks data resulted in an R^2^ of 0.063 and a Bayesian Information Criterium (BIC) of -119.8, which was a poor fit on the data. Applying a piecewise linear regression with one break resulted in a most ideal break at illumination of 11 lux, which resulted in a much better R^2^ of 0.508 and BIC of -180.2, see **Supplementary Figure 3A**. This indicates that performing the test at conditions below 11 lux can have a detrimental effect on the test score.

To determine whether there was a maximum acceptably illuminance we also applied a piecewise linear regression with two breaks on the data. The second break was at an illumination of 1395 lux. This extra break only had a very small effect on R^2^ of 0.511 and the BIC slightly increased to -174.2, indicating overfitting. It does seem to indicate that performing the test around 1395 lux provides ideal conditions for performing SpotChecks, as can be seen in **Supplementary Figure 3B**. There was a minor increase in score as illumination increases from 11 lux until 1395, after which it decreases again. Though, only very few people were tested at more than 1395 lux (*n*=17), so this result is possibly unreliable. Additionally, since the BIC increases from one to two breakpoints, the second breakpoint is likely overfitting on the data so the model with one breakpoint (which has the lowest BIC) would be considered best.


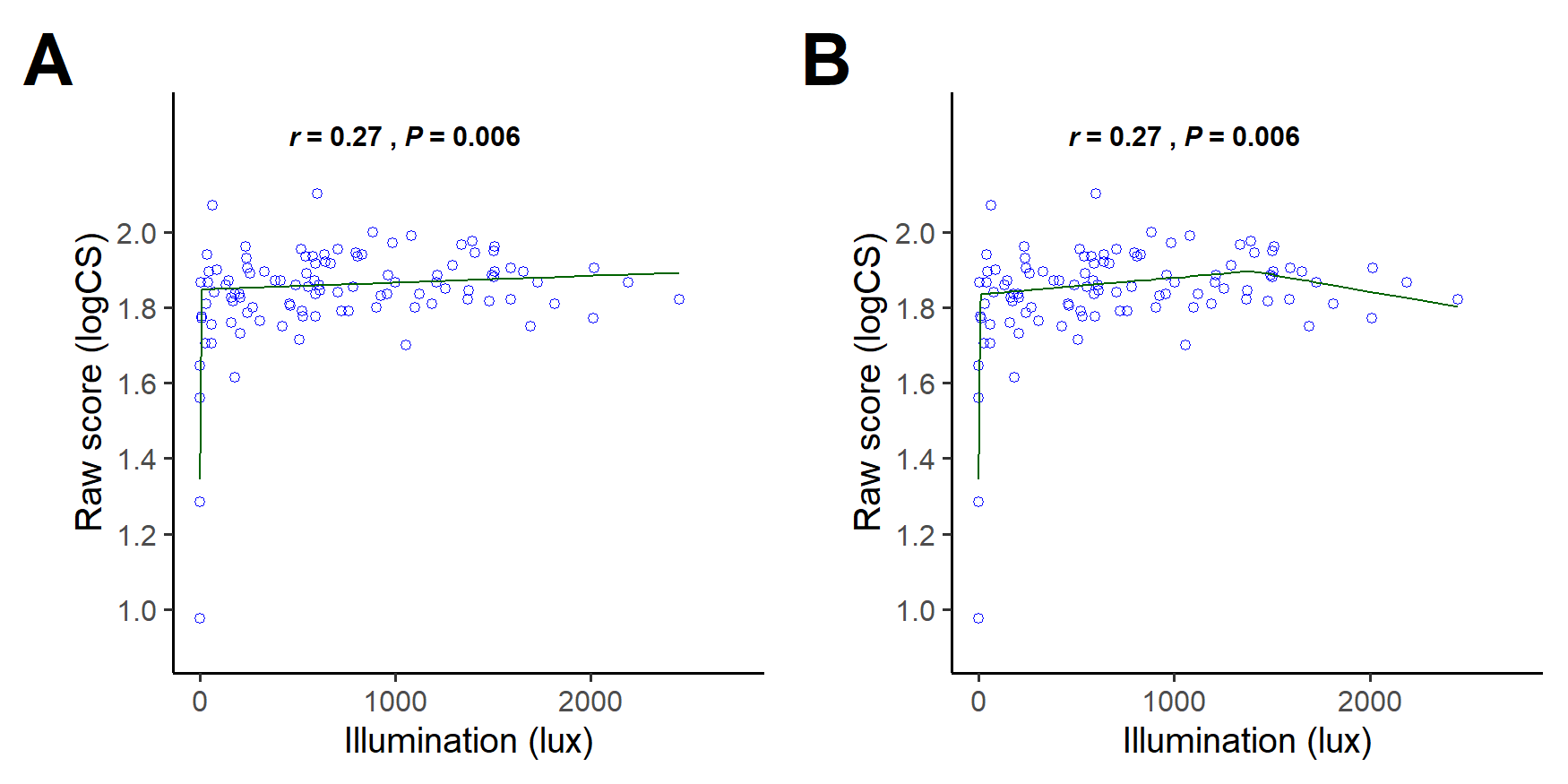


***Supplementary Figure 3: Figures of the piecewise regression for the resilience to illumination.*** *Scatterplot of the illumination vs the mean score on SpotChecks, the green line indicates the piecewise linear regression with breaks at 11 lux (****A****). Scatterplot* ***B*** *is the same as* ***A*** *but with an additional break at 1395 lux. Numerical values indicate Pearson correlation coefficients.*

**II.3 Resilience to participant motivation**

*II.3.1 Participant feedback*

This section is supplementary to section 3.5.1 in the main text and contains the figures of the resilience to the participant feedback. In brief, **Supplementary Figure 4** shows the effect of participant feedback (ranging from 5, extremely negative, to 25, extremely positive) on the raw score, repeatability, and duration of both CS tests.


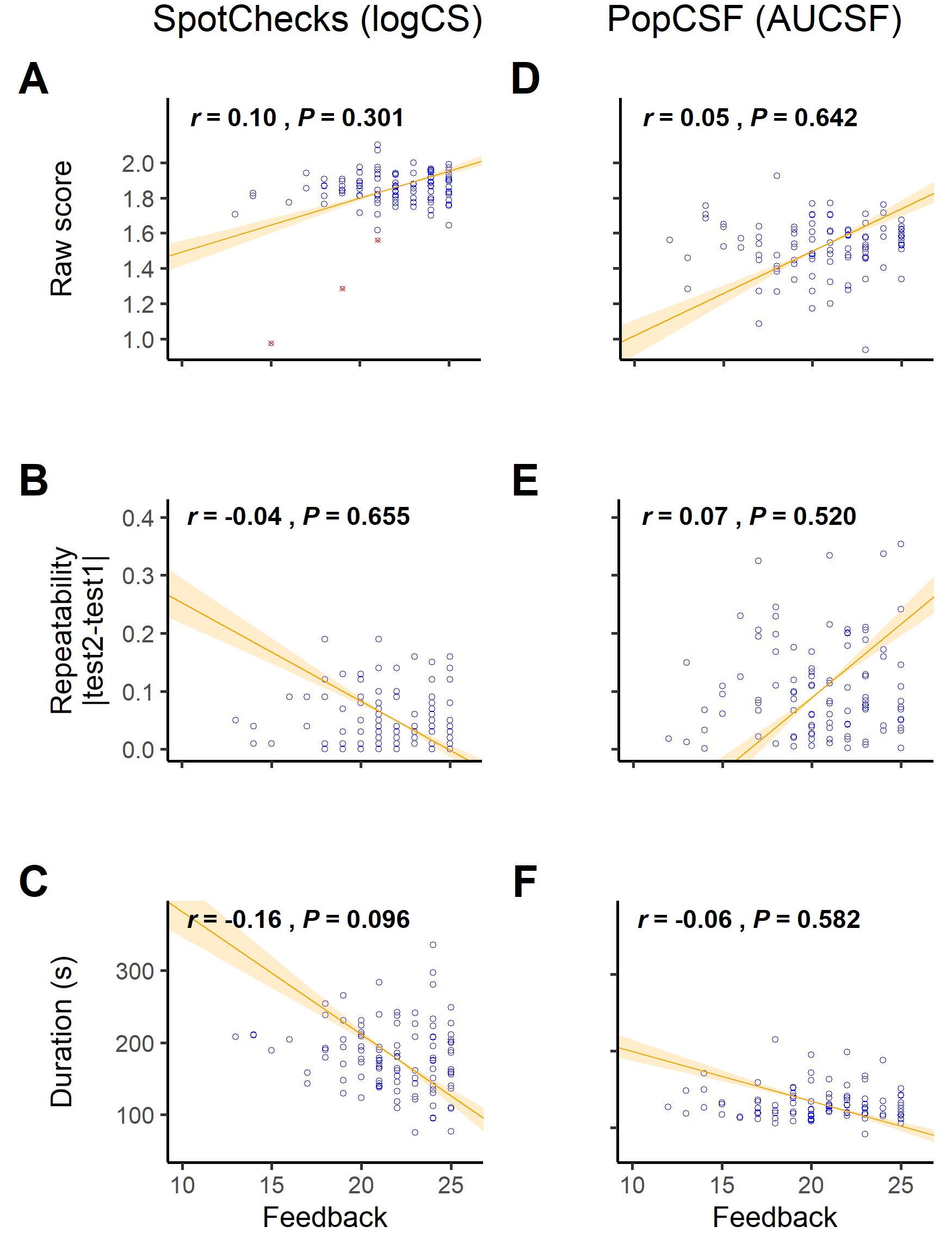


***Supplementary Figure 4: Figures of the resilience to participant feedback.*** *Scatterplots of the raw score vs the feedback score for SpotChecks (****A****), note that in this figure three participants (tested at ≤1lux; shown as a red crossed circle) were removed, and PopCSF (****D****). Scatterplots of the absolute difference between the second and first test vs the feedback score for SpotChecks (****B****) and PopCSF (****E****). Scatterplots of the duration of SpotChecks vs the feedback score for SpotChecks (****C****) and PopCSF (****F****). Numerical values indicate the Pearson correlation coefficient. The line signifies the standard major axis regression, with shaded regions indicating the slope’s 95% confidence interval.*

*II.3.2 HVF reliability*

This section is supplementary to section 3.5.2 in the main text and contains the figures of the resilience to the HVF reliability score. In brief, **Supplementary Figure 5** shows the effect of the HVF reliability metric (the percentage of false positives, false negatives and fixation losses of a visual field test added together) on the raw score, repeatability, and duration of both CS tests.


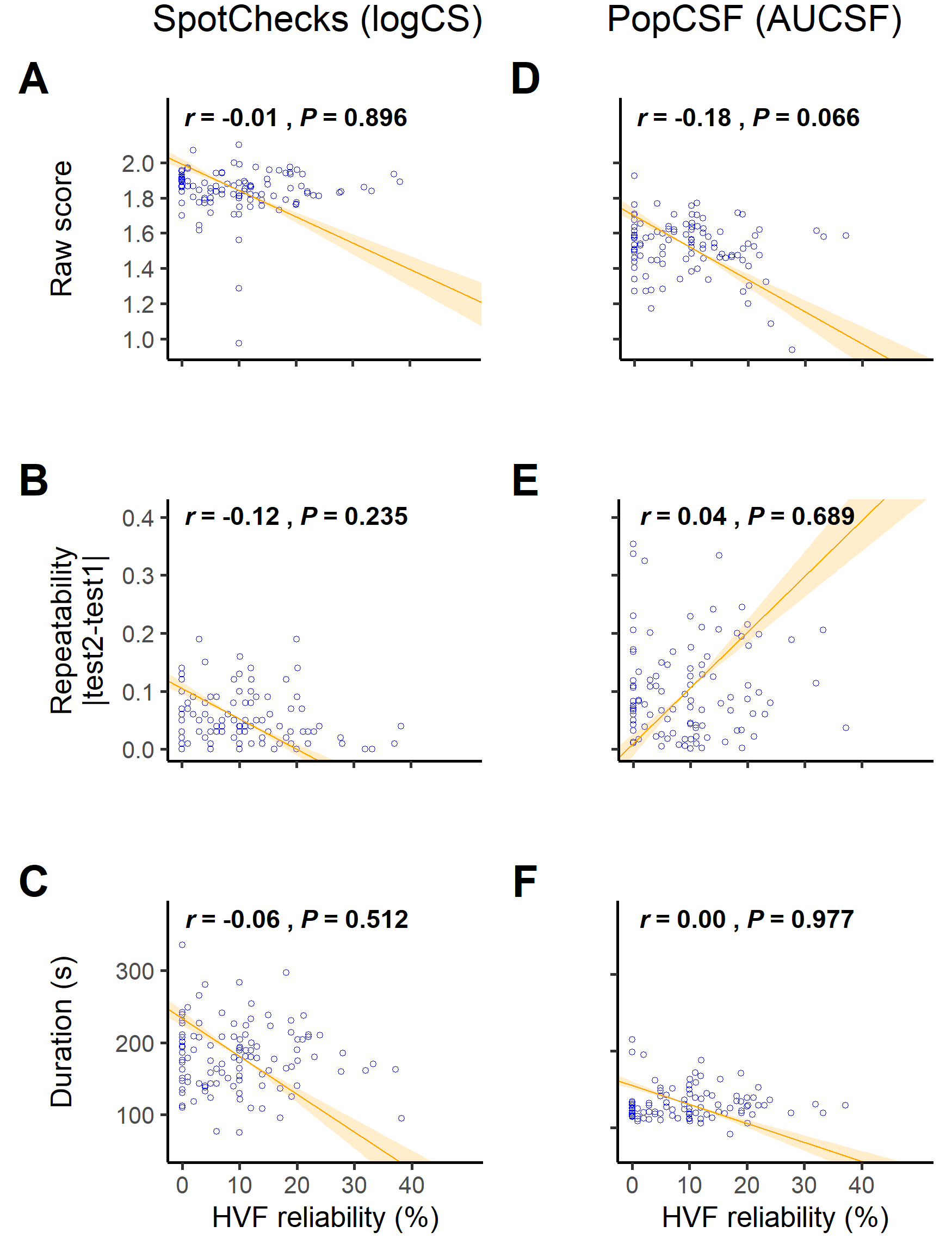


***Supplementary Figure 5: Figures of the resilience to the HVF reliability metric.*** *Scatterplot of the mean SpotChecks score (****A****) and the mean PopCSF score (****B****) with on the x axis the HVF reliability score. Scatterplot of the absolute difference between the two SpotChecks scores (****C****) and the absolute difference between the two PopCSF scores (****D****) with on the x axis the HVF reliability score. Scatterplots of the duration of SpotChecks (****E****) and PopCSF (****F****) with on the x axis the HVF reliability score. Numerical values indicate Pearson correlation coefficients. The line signifies the standard major axis regression, with shaded regions indicating the slope’s 95% confidence interval.*
